# An exposome connectivity paradigm for the mechanistic assessment of the effects of prenatal and early life exposure to metals on neurodevelopment

**DOI:** 10.3389/fpubh.2022.871218

**Published:** 2023-01-09

**Authors:** Ourania Anesti, Nafsika Papaioannou, Catherine Gabriel, Achilleas Karakoltzidis, Vazha Dzhedzheia, Ioannis Petridis, Antonios Stratidakis, Mike Dickinson, Milena Horvat, Janja Snoj Tratnik, Aristidis Tsatsakis, Spyros Karakitsios, Dimosthenis A. Sarigiannis

**Affiliations:** ^1^HERACLES Research Center on the Exposome and Health, Center for Interdisciplinary Research and Innovation, Aristotle University of Thessaloniki, Thessaloniki, Greece; ^2^Centre of Toxicology Science and Research, School of Medicine, University of Crete, Heraklion, Greece; ^3^Environmental Engineering Laboratory, Department of Chemical Engineering, Aristotle University of Thessaloniki, Thessaloniki, Greece; ^4^Science, Technology, and Society Department, Istituto Universitario di Studi Superiori (IUSS), University School for Advanced Study, Pavia, Italy; ^5^Fera Science Ltd., York, United Kingdom; ^6^Department of Environmental Sciences, Josef Stefan Institute, Ljubljana, Slovenia

**Keywords:** exposome, early life exposure, metabolomics, metals, neurodevelopmental disorders

## Abstract

The exposome paradigm through an integrated approach to investigating the impact of perinatal exposure to metals on child neurodevelopment in two cohorts carried out in Slovenia (PHIME cohort) and Greece (HERACLES cohort) respectively, is presented herein. Heavy metals are well-known neurotoxicants with well-established links to impaired neurodevelopment. The links between in *utero* and early-life exposure to metals, metabolic pathway dysregulation, and neurodevelopmental disorders were drawn through urinary and plasma untargeted metabolomics analysis, followed by the combined application of *in silico* and biostatistical methods. Heavy metal prenatal and postnatal exposure was evaluated, including parameters indirectly related to exposure and health adversities, such as sociodemographic and anthropometric parameters and dietary factors. The primary outcome of the study was that the identified perturbations related to the TCA cycle are mainly associated with impaired mitochondrial respiration, which is detrimental to cellular homeostasis and functionality; this is further potentiated by the capacity of heavy metals to induce oxidative stress. Insufficient production of energy from the mitochondria during the perinatal period is associated with developmental disorders in children. The HERACLES cohort included more detailed data regarding diet and sociodemographic status of the studied population, allowing the identification of a broader spectrum of effect modifiers, such as the beneficial role of a diet rich in antioxidants such as lycopene and ω-3 fatty acids, the negative effect the consumption of food items such as pork and chicken meat has or the multiple impacts of fish consumption. Beyond diet, several other factors have been proven influential for child neurodevelopment, such as the proximity to pollution sources (e.g., waste treatment site) and the broader living environment, including socioeconomic and demographic characteristics. Overall, our results demonstrate the utility of exposome-wide association studies (EWAS) toward understanding the relationships among the multiple factors that determine human exposure and the underlying biology, reflected as omics markers of effect on neurodevelopment during childhood.

## Introduction

The connectivity approach to environmental health builds upon the connectivity across different biological scales in a systems biology approach to elucidate the mechanisms underlying the environmental burden of disease and adopting a data-driven paradigm guided by systems biology principles; this approach couples comprehensiveness in exposome and health associations and biological plausibility.

In 2005, a new term was introduced by Wild (1) that addressed the totality of environmental exposures across the complete lifespan, including *in utero* exposure: the exposome. Since, many exposome-related studies have highlighted the importance of perturbation of sensitive biological processes at critical developmental stages and their impact on later life adverse effects (2).

Cumulative exposure, i.e., simultaneous exposure to multiple stressors, is one of the significant exposome aims to address. Although epidemiology usually focuses on identifying associations among single stressors and adverse health outcomes, real-life exposure scenarios are by definition characterized by multiple stressors (3). This implies that the synergistic effect of various stressors, even at low levels, can initiate and sustain perturbations across an adverse outcome pathway (4–6) or even better, across networks of adverse outcome pathways.

The exposome encompasses three exposure sectors, including (a) several external factors (the general ones), (b) targeted external (the specific ones), and (c) internal exposome (7). As a result, exposome analysis includes a broad area of parameters of a completely different nature, such as sociodemographic characteristics, lifestyle, and occupation, and how they define exposure to multiple stressors. On the other hand, the internal exposome includes the biological responses resulting from the multitude of external exposure traits and the genetic heritage of the individual. It is expressed in terms of perturbation in metabolism, oxidative stress and inflammation (8). It is essential to highlight the strong interplay among the parameters above that define the external and internal exposome, and proper interpretation requires a comprehensive multidisciplinary approach.

As a result, assessing the individual exposome requires state-of-the-art analytical and computational methods. Among the developments to evaluate internal exposome, metabolomics comprises a major component. Metabolomics deals with the analysis of all the small molecules delivered by the homeostatic function; thus, they are found in cells, tissues, and biological fluids such as blood and urine. A major advantage of metabolomics compared to other omics techniques such as transcriptomics and proteomics is that it provides a molecular snapshot that is more relevant to phenotypic observations, rendering it an ideal fingerprint of the biological perturbations that are associated with health outcomes observed in large population-based studies. In addition, metabolomics allows the assessment of endogenous and exogenous compounds. Considering the broad chemical spectrum that comprises the metabolome, the efficient coverage of the broad array of metabolites is ensured by the synergistic contribution of various spectrometry methods, where nuclear magnetic resonance (NMR) and liquid chromatography-mass spectrometry are the most important among them (9).

A valid interpretation of the multiple lines of evidence provided by the exposome related methods that generate a large set of heterogeneous data is of utmost importance. A methodology that has proven to be particularly efficient in this direction is the exposome-wide association study (EWAS) approach (10). EWAS builds upon the genome-wide association study (GWAS) paradigm while initially being applied to identify external exposome factors associated with type 2 diabetes (11). However, a significant limitation of this type of studies as they have been applied thus far is that they focus on the pairwise associations between the multiple parameters that define the external exposome and adverse health outcomes. The investigation of the mechanistic explanation between exposure and disease (as described by the internal exposome), remained somehow neglected to date in both epidemiological and toxicological studies (12, 13).

Several studies have dealt with the adverse effects of heavy metals due to their abundant presence in the environment, diet, and consumer products (14), resulting in significant exposure levels to children (15). Heavy metals have caused important health awareness because it has been associated with adverse child neurodevelopment, even at environmentally relevant levels (16). Of particular interest is that cumulative exposure to heavy metals results in effects that go beyond additivity (17, 18).

Given the above, this study aims at providing additional insights on the impact of *in utero* cumulative exposure to phthalates and metals and how they impact child neurodevelopment. Adopting a truly exposomics approach, this is carried out in tandem with the assessment of external factors such as sociodemographic characteristics and diet of the target population and internal exposome factors, as described by the metabolomic signatures. Toward this aim, metabolomics profiles of individuals have been analyzed, starting from human biomonitoring samples. Furthermore, to capture a broader range of metabolites as much as possible, both NMR and liquid chromatography-mass spectromery have been used. Finally, the metabolite signatures have been used to identify statistically significant metabolic pathways for each cohort member; the perturbed pathways identified were associated with the various external exposome factors using the EWAS method.

## Materials and methods

### Cohorts description

#### The PHIME cohort

##### General description

The “Public Health Impact of long-term, low-level Mixed Element exposure in susceptible population strata” (PHIME) study aimed to investigate environmentally relevant exposure to mercury, related to fish and seafood consumption from pregnant mothers, on child neurodevelopment. The study included four Mediterranean countries (Greece, Slovenia, Croatia and Italy), had started in 2006 and concluded in 2011. The study design and recruitment process protocol are described elsewhere (19, 20). Briefly, pregnant women were approached for consent at local health care centers after their ultrasound scan between 20 and 22 gestational weeks, at routine visits between 34 and 38 gestational weeks, or at delivery. Only above 18-year-old expectant mothers carrying a singleton were recruited, having at least 2 years of residency in the areas of interest (without leaving the area during pregnancy for longer than 6 months), and with no history of chronic diseases or abuse of drugs. Aiming at the association between metals and neurodevelopmental toxicity, maternal hair, blood, cord blood, and urine were collected in the 34th week of pregnancy, at or immediately after birth. In addition, breast milk samples were collected 1 month after birth, and at the same time, mothers were asked to complete a questionnaire. Hair samples were stored at room temperature in a zip-lock plastic bag and then analyzed without any cleaning. All other biological samples (maternal blood and cord blood) were stored in a freezer below −20°C. Additional information related to consumer (including smoking) and dietary (with a particular focus on fish and seafood species associated with mercury exposure) behavior of the mother had been included through questionnaires (Table 1).

**Table 1:** Characteristics of the study population (PHIME Cohort), and Bayley scores for cognitive, language and motor Bayley-III scores (29).

**Variable**	**Measure**	**PHIME**	**Sample size (*n*)**
Maternal age at delivery (years)	Mean (SD)	31 (5)	133
Maternal BMI (Kg/m2)	Mean (SD)	25 (5.4)	133
Is the mother smoking?			133
Yes	*n* (%)	24 (18)	
No	*n* (%)	109 (82)	
Maternal education			133
Apprenticeship	*n* (%)	4 (3)	
Secondary school	*n* (%)	38 (28.5)	
High school	*n* (%)	22 (16.5)	
University	*n* (%)	52 (39)	
Master or PhD	*n* (%)	17 (13)	
Mother's employment			133
Employed (full time)	*n* (%)	130 (97.7)	
Employed (part timke time)	*n* (%)	3 (0.3)	
Paternal age at delivery (years)	Mean (SD)	30.9 (9.7)	131
**Is the father/partner smoking?**	
Yes	*n* (%)	24 (19)	
No	*n* (%)	104 (81)	
Paternal education			131
Primary school	*n* (%)	3 (2.2)	
Apprenticeship	*n* (%)	14 (10.7)	
Secondary school	*n* (%)	36 (27.5)	
High school	*n* (%)	46 (35.1)	
University	*n* (%)	25 (19.1)	
Master or PhD	*n* (%)	7 (5.4)	
Partner's employment			132
Employed (full time)	*n* (%)	125 (94.7)	
Employed (partial time)	*n* (%)	2 (1.5)	
Unemployed	*n* (%)	5 (3.8)	
Marital status			131
Married/in relationship	*n* (%)	125 (95)	
Widow	*n* (%)	1 (0.7)	
Divorsed	*n* (%)	7 (4.3)	
Child gender			133
Female	*n* (%)	63 (37)	
Male	*n* (%)	70 (53)	
Breastfeeding			133
No	*n* (%)	5 (4)	
Yes	*n* (%)	128 (96)	
Cognitive score	Mean (SD)	115.6 (13.4)	131
Language score	Mean (SD)	106.6 (12.7)	131
Motor score	Mean (SD)	107.2 (9.3)	131
Fine motor score (FM)	Mean (SD)	12.3 (2.1)	131
Gross motor score (GM)	Mean (SD)	10.1 (1.6)	131
Full scale intelligence quotient (FSIQ)	Mean (SD)	110.1 (12.8)	133

##### Data acquisition

###### Exposure factors

All analyses of THg and MeHg in biological samples were performed at the JoŽef Stefan Institute, Ljubljana, Slovenia. Total Hg (THg) levels were measured in the maternal hair, venous, and cord blood using Direct Mercury Analyzer (21, 22). Cold vapor atomic absorption spectrometry (CVAAS) measured the THg levels in breast milk samples collected 1 month after birth. MeHg in hair was determined by solvent extraction and gas chromatography electron capture detection (GC-ECD). The method has been described elsewhere (23, 24). MeHg in cord blood and milk was determined by acid dissolution, solvent extraction, aqueous phase ethylation, isothermal GC and cold vapor atomic fluorescence detection (CVAFS). A complete description of the method has been given in previously published studies (25, 26). Metals and metalloids (Fe, Mg, Ca, Pb, Mn, Cd, As, Se, Cu, Zn) were determined in blood, cord blood, breast milk and urine samples using inductively coupled plasma mass spectrometry (ICP-MS) as described in the references (27, 28).

###### Exposure and effect modifiers

Toward a comprehensive exposome analysis, additional data on factors that modify either exposure or effect were collected through questionnaires. Eighteen months after delivery, a supplemental questionnaire was administered to report on the children's dietary habits and significant development milestones. These data were related to (a) sociodemographic factors, such as socioeconomic status of the family, educational and marital status of the mother, and child attendance of day-care center until the age of two, (b) mother and child physiological factors such as body weight, height, age of mother at delivery, birth weight and gender of the child, and (c) other parameters such as mode of delivery and breastfeeding.

###### Health outcomes

The neurodevelopment progress of children had been evaluated on month 18 after birth by trained psychologists. For the assessment, Bayley Scales of Infant and Toddler Development, Third Edition, Screening Test (BSID-III) had been used, including (a) cognitive, (b) language, and (c) motor evaluation. For the overall assessment, scaled, as well as composite scores have been accounted for. Finally, children were also examined for autism using the Modified Checklist For Autism in Toddlers (M-CHAT), which was performed by the same psychologists and pediatricians who administered the BSID, and on the same day (19).

#### HERACLES greek cohort

The HERACLES Greek cohort focused on the impacts of exposure to heavy metals (originating from a waste disposal site) on child neurodevelopment in Athens, Greece. The study was initiated in 2012, and 300 children aged 3 to 8 were enrolled, who lived in the proximity of the waste management site (landfill, children with a residential address up to a distance of 12 km were enlisted). Characteristics of the study population including demographics and health outcomes are given in Table 2.

**Table 2:** Characteristics of the study population (HERACLES Cohort), and Bayley scores for cognitive, language and motor Bayley-III scores.

**Variable**	**Measure**	**HERACLES**	**Sample size**
Child gender			299
Female	*n* (%)	138 (46)	
Male	*n* (%)	161 (54)	
Age of mother at birth	Mean (SD)	31.7 (5)	299
Mother's education according to the European Qualifications Framework	Mean (SD)	2.9 (1.1)	298
Level 1: Secondary educational diplomas	*n* (%)	10 (3)	
Level 2: Secondary educational diplomas	*n* (%)	112 (37.6)	
Level 3: Secondary educational diplomas	*n* (%)	116 (40)	
Level 4: Secondary educational diplomas	*n* (%)	10 (3)	
Level 5: Diplomas of higher education and further education, foundation degrees and higher national diplomas	*n* (%)	49 (16)	
Level 6: Bachelor	*n* (%)	1 (0.4)	
Father's education according to the European Qualifications Framework			295
Level 1: Secondary educational diplomas	*n* (%)	10 (3)	
Level 2: Secondary educational diplomas	*n* (%)	131 (45)	
Level 3: Secondary educational diplomas	*n* (%)	112 (38)	
Level 4: Secondary educational diplomas	*n* (%)	5 (1.6)	
Level 5: Diplomas of higher education and further education, foundation degrees and higher national diplomas	n (%)	37 (12.4)	
Childrens' SES index			299
Low	*n* (%)		
Medium	*n* (%)		
High	*n* (%)		
Stress events index (calculated)	Mean (SD)	40.9 (25.7)	299
Breestfeeding	Mean (SD)	2.3 (1.6)	299
BMI	Mean (SD)	18.2 (3.7)	216
Intelligence quotient score (IQ)	Mean (SD)	104.7 (15.1)	279
Verbal comprehension index (CV)	Mean (SD)	107.6 (15.8)	246
Perceptual reasoning index (PRI or RP)	Mean (SD)	105.2 (14.8)	277
Working memory index (WMI or ML)	Mean (SD)	99 (14.3)	258
Processing speed index (PSI)	Mean (SD)	101.9 (13.6)	268

##### Data used in the analysis

###### Exposure factors

Exposure to heavy metals was assessed to understand the association between neurodevelopmental progress and environmental factors. More specifically, As, Hg and Cd in urine, Pb in blood and Hg and Mn in hair were analyzed. To better understand the totality of exposures related to the waste management site, residential distance from the landfill, as well as concentration of heavy metals in the soil at the home of the children were taken into account. Details on the study design, as well as the chemical and metabolomics analysis are given elsewhere (30).

###### Exposure and effect modifiers

Similarly to the PHIME study, exposure and effect modifiers were also included. Namely (a) sociodemographic factors (including socioeconomic status, mother and father education, as well as stress events), (b) child physiology factors, including body mass index and gender, and (c) dietary parameters such as breastfeeding, and description of dietary components in detail, including meat products (e.g., pork meat, beef, lamb, sausages), fish and other seafood, poultry products (eggs, chicken), dairy products (milk, yogurt), nuts, fruits, vegetables and snacks (biscuits, chocolates) and (d) concentration of Se in the mother during all perinatal period. The above-mentioned dietary data have been collected using Food Frequency Questionnaires (FFQ).

###### Health outcomes investigated

The assessment of children (aged 3–8 years) neurodevelopmental disorders has been carried out using the following test batteries, namely (a) the Child Behavior Checklist, (b) the Cambridge Neuropsychological Test Automated Battery—CANTAB (31, 32), (c) the Social Responsiveness Scale—SRS which is used primarily to measure Autism Spectrum Disorders (ASD) severity (33, 34) and (d) the Wechsler Intelligence Scale for Children—Fourth Edition for general intelligence. CANTAB was used for the assessment of cognitive development, while Wechsler Intelligence Scale provided information on the intelligence quotient (IQ) or mental development index (MDI). All the resulting scores were considered as outcomes. The resulting scores were included in the downstream EWAS analysis, to examine the effects of exposure to metals on cognition, motor, and social behavior in a comprehensive manner.

### Metabolomics analysis

#### UPLC-HRMS

For UPLC-HRMS analysis, urine and plasma samples were thawed under stable conditions to standard protocols described from Want et al. (35) and Theodoridis et al. (36).

Urine: 600 μl from urine samples was centrifuged at 10,000 rpm for 10 min. A supernatant of 500 μl was placed on autosampler vials and diluted with 1:2 of LC-MS water. After that, the samples were ready for analysis. The autosampler operated at 4°C.

Plasma: A quantity of 200 μl plasma sample was transferred to a new Eppendorf and diluted with 600 μl of cold methanol. After that, the samples were centrifuged at 10,000 rpm for 20 min. A supernatant of 300 μl was placed to a clean Eppendorf and dried using a Techne Sample Concentrator. All samples were reconstructed with 100 μl of LC-MS water. The samples were further centrifuged (10,000 rpm for 20 min). A final quantity of 95 μl of supernatant was placed into 2-ml vials with inserts and placed in UPLC autosampler operating at 4°C, ready for analysis. In addition, a 50 μl quantity was used for the pooled quality control (QC) sample.

Sample analysis was performed on a ThermoFisher Scientific model LTQ Orbitrap Discovery MS, with a resolution of 30,000. The spectra from both urine and plasma samples were acquired in both positive and negative ionization modes. The mass scanning range was set at 50–1,000 m/z. The capillary temperature was set at 320°C. Nitrogen sheath gas and auxiliary gas was flow rate was set to 40 L/min and 8 L/min respectively and the spray voltage at 4.5 kV. LC-MS uses a gradient of two solvents. One hundred percentage LC-MS water with 0.1% formic acid as mobile phase A and 100% methanol with 0.1% formic acid as mobile phase B. For urine samples analysis, the flow rate was 500 μl/min for both the positive and negative modes. For the plasma sample analysis, the flow rate was 400 μl/min in positive mode and 360 μl/min in the negative mode.

For chromatographic separation, an Acquity UPLC HSS T3 column (100 × 2.1 mm, 1.8 μm, Waters, Milford, MA, USA) was used, which was kept at a constant temperature of 40°C. The gradient of the mobile phase for urine samples for both positive and negative mode was: 1% B at 0 min, 1% B at 1 min, 15% B at 3 min, 50% B at 6 min, 95% B at 9 min, 95% B at 10 min, 1% B at 10.1 min and 1% B at 14 min. For the plasma samples, a different gradient was used; for the positive mode: 0% B at 0 min, 0% B at 1 min, 100% B at 16 min, 100% B at 20 min, 0% B at 22 min, 0% B at 24 min. For the negative mode: 0% B at 0 min, 0% B at 2 min, 100% B at 17 min, 100% B at 22 min, 0% B at 24 min, 0% B at 26 min. Blank samples, and pooled QC samples, were used to monitor the systematic signal deviations between the batches. Two blank samples containing internal standards in known concentrations were injected before each batch, in the middle and at the end of each batch, for (a) checking the condition of the column and (b) calculating the mass error in ppm of the isotope patterns. Additional, ten QC pooled samples were injected at the onset of the experiment for the conditioning of the column of the LC system. The pooled QC sample was repeated every ten samples in urine samples analysis, whereas plasma samples the QC samples were repeated every five plasma samples.

#### NMR

First samples were centrifuged at 10,000 rpm for 10 min. Then, 500 μL of the supernatant was transferred to a new Eppendorf and mixed with 120 μL of buffer solution (Na_2_HPO_4_,0.2 M, NaH_2_PO_4_,0.3 Min 50% D_2_O/50% H_2_O) and 0.1% TSP-d4, which was used as chemical shift reference (δH 0.00 ppm). The pH of the samples was 7.337. All samples were vortexed and placed in −4°C for 7 min. After 7 min, samples were thawed and centrifuged, and the supernatant was transferred to a 5 mm NMR tube. A 1 ml aliquot was evaporated to dryness under vacuum for plasma samples. Reconstitution was performed with 50 μl deuterium oxide (D_2_O), followed by vortex and centrifugation at 14,000 rpm for 5 min at 4°C. The supernatant was evaporated once more and then reconstructed in 660 μl of 100 mM phosphate buffer containing 1 mM trimethylsilyl propanoic acid (TSP) and 1 mM sodium azide. The extract was vortexed before a final centrifugation step at 14,000 rpm for 5 min at 4°C. The supernatant was transferred to a 5 mm NMR tube.

For urine samples analysis, a 600 MHz Varian spectrometer was used. The spectrometer frequency was 599.938 MHz in an OneNMR Probe and a ProTune System (Agilent) using on-resonance pre-saturation to suppress the intensity of the water signal. Proton chemical shifts typically range from −2 to 10 ppm (spectral width 9615.4 Hz), with 128 scans, a relaxation delay of 2 s, acquisition time 4 s and pulse width 8.587 μs. Plasma samples were acquired in a Bruker Avance 500 MHz NMR spectrometer equipped with a TCI cryoprobe. Data acquisition and processing were performed using the software package Topspin v 1.3 (Bruker, Germany). The central frequency used was 500.1323505 MHz, using on-resonance pre-saturation to suppress the intensity of the water signal, followed by a 1D NOESY pulse sequence with irradiation of the residual water signal the mixing time (200 ms). The observation pulse length was set at 10.0 μs, the delay between transients was 3 s, and 65,536 complex data points were acquired with a spectral width of 10,400 Hz (corresponding to a chemical shift range of 14.0019 parts per million, ppm), giving a final acquisition time of 4.679 s. The total time of the experiment was ~67 min and included eight unrecorded (dummy) transients and 512 acquisition transients (scans).

### Data analysis

#### Spectra preprocessing

Raw data generated from positive and negative ionization were pre-processed as two different experiments. The tool msConvert included in the ProteoWizard toolkit (37, 38) was used to translate the data into the.mzML open format. Spectral processing was performed using the Bioconductor R—based packages XCMS v.3.10.1 (39) running under R version 3.6.1 (https://www.r-project.org/). Chromatographic peak detection was performed using the centWave algorithm, and the Obiwarp method was used for alignment. The *definitions* function was used for peak correspondence. We used the fillChromPeaks method to fill in intensity data for missing values from the original files due to false negatives. Finally, the *PerformPeakAnnotation* function is used for isotope and adduct annotation using the CAMERA package (40). The resulting matrix was further reduced by the 80% rule applied to the QC samples to obtain consistent variables. The instrument and overall process variability were then determined by calculating the median RSD for authentic internal standards and all endogenous metabolites. Normalization by the median, mean centering scaling, and log transformation was performed to transform the data matrix into a more Gaussian-type distribution, thus reducing systematic error in experimental conditions. The annotation R package xMSAnnotator (41) was used to perform the accurate mass carries in online compound databases [HMDB (42), LipidMaps (43), and KEGG (44)]. The adduct list used for database matching included “M+2H,” “M+H+NH4,” “M+ACN+2H,” “M+2ACN+2H,” “M+H,” “M+NH4,” “M+Na,” “M+ACN+H” in positive ionization mode, and the following in negative ionization mode: “M+ACN+Na,” “M+2ACN+H,” “2M+H,” “2M+Na,” “2M+ACN+H” “M-2H,” “M-3H,” “M-H2O-H,” “M+Na-2H,” “M-H,” “M+Cl,” “M+FA-H,” “M+K-2H,” “2M-H.” The list of the detected candidate metabolites was filtered by metabolite status (Detected) and biospecimen (Blood). Confirmation of identified biomarkers was performed by comparing the RT and fragmentation pattern of authentic analytical standards from the in-house library or MS/MS spectra available in databases like HMDB and Metlin (45).

Spectral analysis of NMR data proceeded using MestReNova (Mnova 11.0.3) (http://mestrelab.com), while for metabolite identification ChenoMx (http://www.chenomx.com) was used in addition. Briefly, after loading the spectra of all the samples using the superimposed command, the first step was to correct the position of the reference peak sample. The detected peaks (binning) were grouped using length values lower than 0.04 ppm in this study; the used reference was deuterium oxide (D_2_O) due to the used buffer. After the reference correction, the alignment of all reference peaks was checked. The Smoother Whittaker algorithm was chosen for baseline correction; after that, the spectrum phase was checked and corrected. For phase correction, an automatic algorithm is preferable. The detected peaks (binning) were grouped using length values lower than 0.04 ppm. Then, the spectrum was imported into the ChenomX NMR Suite 8.2 for peak identification. Finally, the previously identified peaks of TSP and metabolites were integrated using the MNOVA software, and the metabolite peaks were identified. In cases where multiple peaks and peaks characterized a metabolite in different areas of ppm, the area of all these peaks were added to fill the corresponding cell on the sheet of import file to MPP.

All LC-MS and NMR data were deposited to the EMBL—EBI MetaboLights database with the MTBLS1882 identifier.

#### Pathway analysis

Pathway analysis has been carried out by combining two GeneSpring modules, Mass Profiler Professional (MPP) and Pathway Architect (Agilent Technologies). To calculate the probability of enriching the listed metabolites with a pathway, a hypergeometric test was used, while the *p*-values have been corrected for multiple testing using the Benjamin-Hochberg method (46).

#### EWAS

The exposome-wide association study (EWAS) paradigm has been coined Patel et al. (11) to describe the associations among several variables composing the human exposome, using unsupervised learning (47). The study predictors can be classified in numerical, nominal categorical, or ordinal categorical. For example, the exposure factors were continuous variables since they were data from mass spectrometry. In contrast, the variables regarding the frequency of specific food items or water consumption were ordinal. Before applying the logistic regression algorithm, we investigated the skewness of the continuous variables and then transformed them into logarithmic form. Next, we adjusted each observation to the mean and scaled it by the standard deviation and after that it was classified into bins. The number of selected bins is 10. In the present study the range of each class is calculated taking the difference between the maximum and minimum value divided by the number of bins. Therefore, observations are clustered /into categorical classes so that they can then be used in logistic regression. The rest of health outcome variables that were used are presented in the following table in order to simply describe the limits of each bin. In addition, we converted categorical variables into numerical ones, using integer encoding, whereby each unique label was mapped to an integer (Table 3).

**Table 3:** Conversion of continuous variables into categorical ones.

**Variable**	**Bin 1**	**Bin 2**	**Bin 3**	**Bin 4**	**Bin 5**	**Bin 6**	**Bin 7**	**Bin 8**	**Bin 9**	**Bin 10**
CBC9_1	(0, 0.4)	(0.4, 1)	(1, 1.6)	(1.6, 2.7)	(2.7, 4.1)	(4.1, 6.2)	(6.2, 9.1)	(9.1, 13.2)	(13.2, 18.9)	(18.9, 28)
CBC9_2	(33, 36)	(36, 39.2)	(39.2, 41.9)	(41.9, 45.6)	(45.6, 49.7)	(49.7, 54.1)	(54.1, 57.8)	(57.8, 62.9)	(62.9, 68.5)	(68.5, 76)
CBC9_3	(5, 6.9)	(6.9, 9.5)	(9.5, 12.9)	(12.9, 17.3)	(17.3, 23.3)	(23.3, 31.1)	(31.1, 41.5)	(41.5, 55.2)	(55.2, 73.3)	(73.3, 100)
CBC10_1	(0, 0.5)	(0.5, 1)	(1, 1.9)	(1.9, 2.9)	(2.9, 4.7)	(4.7, 7.4)	(7.4, 10.4)	(10.4, 15.6)	(15.6, 23.4)	(23.4, 33)
CBC10_2	(33, 35.8)	(35.8, 39)	(39, 42.4)	(42.4, 46.2)	(46.2, 50.2)	(50.2, 54.7)	(54.7, 59.5)	(59.5, 63.7)	(64.7, 69.3)	(69.3, 77)
CBC10_3	(5, 7)	(7, 9.5)	(9.5, 12.7)	(12.7, 16.8)	(16.8, 23.8)	(23.8, 31.3)	(31.3, 41.2)	(41.2, 53.9)	(53.9, 70.6)	(70.6, 100)
CBC19_1	(0, 0.3)	(0.3, 0.7)	(0.7, 1.3)	(1.3, 2.1)	(1.8, 2.8)	(2.8, 4)	(4, 5.7)	(5.7, 7.9)	(7.9, 10)	(10, 15)
CBC19_2	(50, 52.3)	(52.3, 55.1)	(55.1, 57.5)	(57.5, 60.5)	(60.5, 63.1)	(63.1, 66.5)	(66.5, 69.3)	(69.3, 73)	(73, 76.1)	(76.1, 81)
CBC19_3	(50, 53.8)	(53.8, 57.7)	(57.7, 61.9)	(61.9, 64.9)	(64.9, 69.6)	(69.6, 74.7)	(74.7, 80.2)	(80.2, 86)	(86, 92.3)	(92.3, 100)
CBC20_1	(0, 1)	(1, 2)	(2, 3)	(3, 4)	(4, 5)	(5, 6)	(6, 7)	(7, 8)	(8, 9)	
CBC20_2	(50, 52.8)	(52.8, 54.6)	(54.6, 57)	(57, 58.9)	(58.9, 61.5)	(61.5, 63.6)	(63.6, 66.3)	(66.3, 68.6)	(68.6, 71.5)	(71.5, 74)
CBC20_3	(50, 54)	(54, 57.4)	(57.4, 60.9)	(60.9, 66)	(66, 70.1)	(70.1, 76)	(76, 80.7)	(80.7, 85.7)	(85.7, 92.8)	(92.8, 100)
CBC21_1	(0, 0.3)	(0.3, 0.7)	(0.7, 1.2)	(1.2, 1.9)	(1.9, 2.7)	(2.7, 3.8)	(3.8, 5.3)	(5.3, 7.1)	(7.1, 9.5)	(9.5, 13)
CBC21_2	(50, 52)	(52, 53.8)	(53.8, 56.2)	(56.2, 58.2)	(58.2, 60.8)	(60.8, 62.9)	(62.9, 65.7)	(65.7, 68)	(68, 71)	(71, 75)
CBC21_3	(50, 53)	(53, 57.9)	(57.9, 61.7)	(61.7, 65.9)	(65.9, 70.3)	(70.3, 75)	(75, 81.8)	(81.8, 87.3)	(87.3, 93.1)	(93.1, 100)
TRF9_1	(0, 0.5)	(0.5, 1.2)	(1.2, 2.3)	(2.3, 3.7)	(3.7, 5.9)	(5.9, 9)	(9, 13.6)	(13.6, 20.3)	(20.3, 30)	(30, 46)
TRF9_2	(37, 40.4)	(40.4, 43.7)	(43.7, 48.1)	(48.1, 53.1)	(53.1, 57.3)	(57.3, 63.2)	(63.2, 68.3)	(68.3, 73.8)	(73.8, 81.2)	(81.2, 89)
TRF9_3	(10, 12.3)	(12.3, 15.5)	(15.5, 19.6)	(19.6, 24.6)	(24.6, 30.8)	(30.8, 38.6)	(38.6, 48.3)	(48.3, 60.3)	(60.3, 75.3)	(75.3, 100)
TRF10_1	(0, 0.4)	(0.4, 1)	(1, 1.8)	(1.8, 3.4)	(3.4, 5.2)	(5.2, 7.7)	(7.7, 11.3)	(11.3, 16.4)	(16.4, 25.8)	(25.8, 38)
TRF10_2	(41, 43.3)	(43.3, 46.6)	(46.6, 49.3)	(49.3, 52.2)	(52.2, 56.1)	(56.1, 59.4)	(59.4, 62.9)	(62.9, 67.6)	(67.6, 71.5)	(71.5, 77)
TRF10_3	(18, 22)	(22, 26.2)	(26.2, 31.2)	(31.2, 37.1)	(37.1, 44.1)	(44.1, 52.3)	(52.3, 62)	(62, 73.6)	(73.6, 87.2)	(87.2, 100)
TRF21_1	(0, 0.4)	(0.4, 0.8)	(0.8, 1.7)	(1.7, 2.6)	(2.6, 4.2)	(4.2, 5.9)	(5.9, 9)	(9, 12.2)	(12.2, 18.3)	(18.3, 27)
TRF21_2	(50, 52.4)	(52.4, 55)	(55, 57.7)	(57.7, 60.6)	(60.6, 63.2)	(63.2, 66.4)	(66.4, 69.7)	(69.7, 73.1)	(73.1, 76.8)	(76.8, 81)
TRF21_3	(50 53.2)	(53.2, 57.4)	(57.4, 61.8)	(61.8, 65.4)	(65.4, 70.4)	(70.4, 75.9)	(75.9, 81.7)	(81.7, 86.4)	(86.4, 93.1)	(93.1, 100)
TRF22_1	(0, 1)	(1, 2)	(2, 3)	(3, 4)	(4, 5)	(5, 6)	(6, 7)	(7, 8)	(8, 9)	(9, 10)
TRF22_2	(50, 51.8)	(51.8, 53.8)	(53.8, 56)	(56, 57.7)	(57.7, 60)	(60, 62.4)	(62.4, 64.4)	(64.4, 67)	(67, 69.6)	(69.6, 73)
TRF22_3	(50, 53.1)	(53.1, 57.7)	(57.7, 61.3)	(61.3, 65.2)	(65.2, 70.8)	(70.8, 75.3)	(75.3, 80.1)	(80.1, 86.9)	(86.9, 92.4)	(92.4, 100)
TRF23_1	(0, 0.3)	(0.3, 0.7)	(0.7, 1.3)	(1.3, 2)	(2, 2.9)	(2.9, 4.2)	(4.2, 5.8)	(5.8, 7.9)	(7.9, 10.7)	(10.7, 15)
TRF23_2	(50, 51.9)	(51.9, 53.8)	(53.8, 55.6)	(55.6, 58)	(58, 60)	(60, 62.1)	(62.1, 64.2)	(64.2, 66.9)	(66.9, 69.2)	(69.2, 73)
TRF23_3	(50, 53.7)	(53.7, 57.8)	(57.8, 61.1)	(61.1, 65.7)	(65.7, 70.6)	(70.6, 75.9)	(75.9, 81.6)	(81.6, 86.2)	(86.2, 92.7)	(92.7, 100)
RVP_1	(1, 1.6)	(1.6, 2.2)	(2.2, 3.2)	(3.2, 4.2)	(4.2, 5.8)	(5.8, 7.6)	(7.6, 10.1)	(10.1, 12.9)	(12.9, 17.1)	(17.1, 23)
SWM_1	(0, 0.6)	(0.6, 1.4)	(1.4, 3)	(3, 5.2)	(5.2, 9.1)	(9.1, 14.7)	(14.7, 23.3)	(23.3, 38.8)	(38.8, 60.7)	(60.7, 101)
SWM_2	(22, 23.8)	(23.8, 25.4)	(25.4, 27.5)	(27.5, 29.5)	(29.5, 31.9)	(31.9, 34.1)	(34.1, 36.9)	(36.9, 39.5)	(39.5, 42.7)	(42.7, 47)
SST_3	(1, 2.4)	(2.4, 4.3)	(4.3, 8.3)	(8.3, 13.8)	(13.8, 22.5)	(22.5, 39.8)	(39.8, 63.8)	(63.8, 112)	(112, 178)	(178, 285)
SST_4	(1, 2.3)	(2.3, 4.2)	(4.2, 7.8)	(7.8, 12.6)	(12.6, 22.2)	(22.2, 35.1)	(35.1, 60.2)	(60.2, 94.2)	(94.2, 161)	(161, 252)
SST_7	(235, 242)	(242, 249)	(249, 256)	(256, 263)	(263, 271)	(271, 279.6)	(279.6, 287)	(287, 296)	(296, 305)	(305, 314)
SOC_13	(1, 1.4)	(1.4, 1.9)	(1.9, 2.4)	(2.4, 3.1)	(3.1, 3.9)	(3.9, 4.8)	(4.8, 5.9)	(5.9, 7.3)	(7.3, 8.8)	(8.8, 12)
SOC_14	(0, 1)	(1, 2)								
PGT_P	(6, 8)	(8, 10.8)	(10.8, 13.7)	(13.7, 18.2)	(18.2, 23)	(23, 30.4)	(30.4, 40)	(40, 50.3)	(50.3, 66)	(66, 84)
PTT_P	(35, 38.2)	(38.2, 41.5)	(41.5, 45.9)	(45.9, 49.9)	(49.9, 54.2)	(54.2, 59.9)	(59.9, 65.1)	(65.1, 70.7)	(70.7, 76.8)	(78.1, 85)
PGT_T	(0, 0.6)	(0.6, 1.5)	(1.5, 2.9)	(2.9, 5.6)	(5.6, 9.2)	(9.2, 14.8)	(14.8, 23.4)	(23.4, 40.3)	(40.3, 62.9)	(62.9, 102)
PTT_T	(1, 1.7)	(1.7, 2.6)	(2.6, 3.8)	(3.8, 5.8)	(5.8, 8)	(8, 10.9)	(10.9, 15.7)	(15.7, 21.2)	(21.2, 28.4)	(28.4, 40)
IQ	(64, 69.3)	(69.3, 75.8)	(75.8, 81.7)	(81.7, 88)	(88, 96.2)	(96.2, 104)	(104, 113)	(113, 122)	(122, 131)	(131, 145)
CV	(64, 69.6)	(69.6, 75.1)	(75.1, 81)	(81, 88.7)	(88.7, 95.7)	(95.7, 103.3)	(103, 113)	(113, 122)	(122, 132)	(132, 146)
RP	(65, 70.4)	(70.4, 75.7)	(75.7, 81.4)	(81.4, 88.8)	(88.8, 95.5)	(95.5, 102.7)	(102.7, 112)	(112, 120)	(120, 129)	(129, 142)
ML	(61, 66)	(66, 72)	(72, 78.7)	(78.7, 84.6)	(84.6, 92.4)	(92.4, 99.4)	(99.4, 109)	(109, 118)	(118, 127)	(127, 140)
VE	(65, 70.4)	(70.4, 76.3)	(76.3, 82.7)	(82.7, 89.6)	(89.6, 98.4)	(98.4, 106.6)	(106.6, 116)	(116, 125)	(125, 136)	(136, 148)

Henceforth, we used survey-weighted logistic regression models to associate each of the exposure factors and modifiers with a health outcome like the psychomotor scores (cognitive, language, and motor score), while adjusting for child gender, and a variety of sociodemographic, anthropometric and other gestational and post-delivery parameters collected from self-reported questionnaires. Spearman correlation, a non-parametric test, was applied to calculate the correlations among variables and avoid any distributional assumptions. The False Discovery Rate (FDR) q-value was calculated to associate the factor with a health outcome, like Bayley test levels, controlling the type I error using the Benjamini-Hochberg step-down approach (48). Applying permutation resampling to Bayley III test scores allowed us to validate the FDR results (49). The significance level was set at 0.05, corresponding to an FDR of 10%. The same procedure was repeated for the study of exposure factors and the detected metabolic pathway associations, as well as for revealing the associations between health outcomes (Bayley III test) and the perturbed metabolic pathways. An Ubuntu 16.04 server was used for carrying out the calculations, and the “X-Wide Association Analyses” R package (50) was used for the logistic regression and the FDR calculations, while the “RCircos” package (51) was employed for visualizing the results through correlation globes. Dataset skewness correctness has been checked using the R library “moments,” while the R package “permute” was used for the restricted permutations of the randomization tests. Finally, for data analysis, clustering, utility operations, computing sample size and power, the Hmisc R package (52) was used to import and annotate datasets, and impute missing values.

In order to generate a list of candidate biomarkers for the stressors of interest, which were identified by untargeted metabolomics analysis, a manual literature search was carried out including combinations of the following terms: “exposure,” “metals,” “neurodevelopment disorders,” “biomarkers” and “underlying mechanisms.” The query was performed for both levels of analysis (molecular biomarkers and pathway analysis) for all the experiments. It was designed not only to determine candidate biomarkers based on their known biological function but also to avoid the exclusion of biomarkers associated with the occurrence of a stressor, even though there is yet lack of knowledge regarding its exact biological function.

## Results

### PHIME cohort

**Figure 1:**
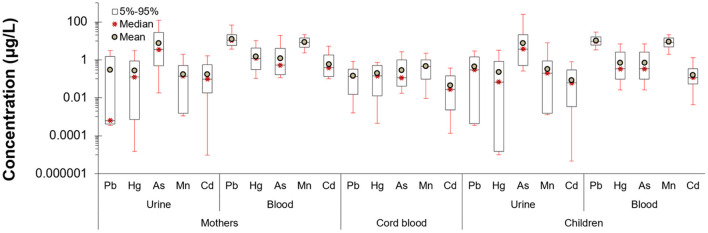
Pregnancy and early life biomonitored levels of heavy metals in blood and urine of PHIME cohort participants (*n* = 160). Mean concentrations of metals are below 1 μg/L, except for arsenic, where the concentration levels are the higher among the rest of the metals. Lead had the higher concentration in blood samples, which is expected given that lead is a compound that bioaccumulates.

The levels of metals in the urine and blood samples of the mothers and the children (as well as the cord blood during the delivery) are presented in Figure 1. For most of the metals in urine, mean concentrations are below 1 μg/L, except for arsenic, where concentration levels are higher. Regarding blood, higher concentrations are observed for lead, an expected feature since lead is bioaccumulative.

**Figure 2:**
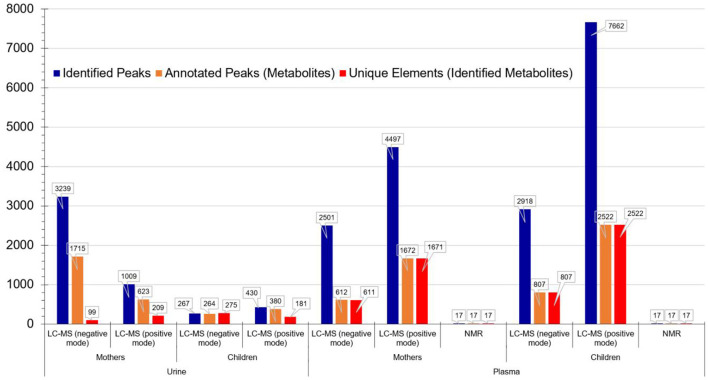
Metabolomics analysis results per matrix, population group and technology for the PHIME cohort. It is evident that the use of different platforms (LC-MS and NMR) resulted in a much broader coverage of detected metabolites.

The corresponding metabolomics results are presented in Figure 2. The use of different platforms (LC-MS and NMR) resulted in a much broader coverage of detected metabolites.

In terms of metabolic pathway analysis, it was found that exposure to metals is negatively correlated with the metabolism of fatty acids, amino and nucleotide sugar, porphyrin and chlorophyll, and glycolysis/gluconeogenesis (Table 4). The identified pathways indicate perturbations in mitochondrial respiration. The down-regulated activity of these metabolic pathways resulted in mitochondrial function impairment during early pregnancy (in relation to heavy metals exposure). Considering the importance of mitochondria on energy metabolism and cellular signaling, their dysfunction may very likely result in impaired neurodevelopment (53).

**Table 4:** Identified biomarkers and pathways related to neurodevelopmental adverse outcomes.

**Pathway**	**Identified biomarker**	**Technique**	**Biological fluid**	**Samples from mothers (M)/Children (C)**	**Health outcome**
Urea cycle	L-arginine	LC-MS	Plasma	C	Autism
	L-citrulline	LC-MS	Plasma	C	Autism
Glycolysis/gluconeogenesis	alpha-D-Glucose	LC-MS	Plasma	C	Autism
	(S)-Lactate	LC-MS	Plasma	C	Autism
	Acetate	LC-MS	Plasma	M	
	alpha-D-Glucose	LC-MS	Plasma	M	
	(S)-Lactate	LC-MS	Plasma	M	
Serotonin and melatonin biosynthesis	L-tryptophan	LC-MS	Plasma	C	Autism
	L-tryptophan	LC-MS	Plasma	M	

Exposure to metals is highly correlated with the urea cycle, glycolysis/gluconeogenesis, serotonin and melatonin biosynthesis, due to the presense of l-arginine, l-citrulline, alpha-d-glucose, (s)-lactate, acetate, and l-tryptophan in the participants' blood samples. The identified pathways, indicate perturbations in mitochondrial respiration after exposure to metals.

**Figure 3:**
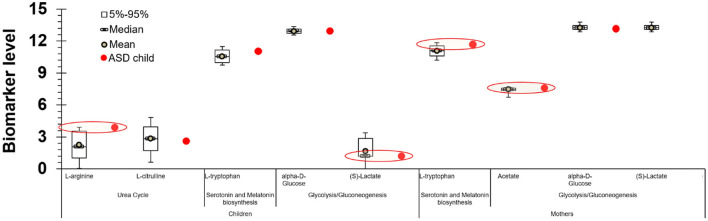
Levels of specific metabolites, heavy metals and for the children with ASD participated in the PHIME cohort. L-arginine of the children diagnosed with ASD, L-tryptophan and acetate of their mothers were above the 95th percentile (S)-Lactate of the children was below the 5th percentile. As and Hg levels in children blood diagnosed with ASD were above the 95th percentile.

Also of particular interest are the results that correlate the levels of specific metabolites with heavy metals and ASD. Among the cohort participants, 11 children had been diagnosed with ASD. L-arginine of the children, L-tryptophan and acetate of their mothers were above the 95th percentile (S)-Lactate of the children was below the 5th percentile. As and Hg levels in children's blood were above the 95th percentile. Even though this result is indicative of a positive association between these biomarkers and ASD diagnosis, the influence of the low sample size on the statistical power of the analysis must be considered. This suggests that the associations between ASD and the identified biomarkers must be further examined with the involvement of larger populations (Figure 3).

**Figure 4:**
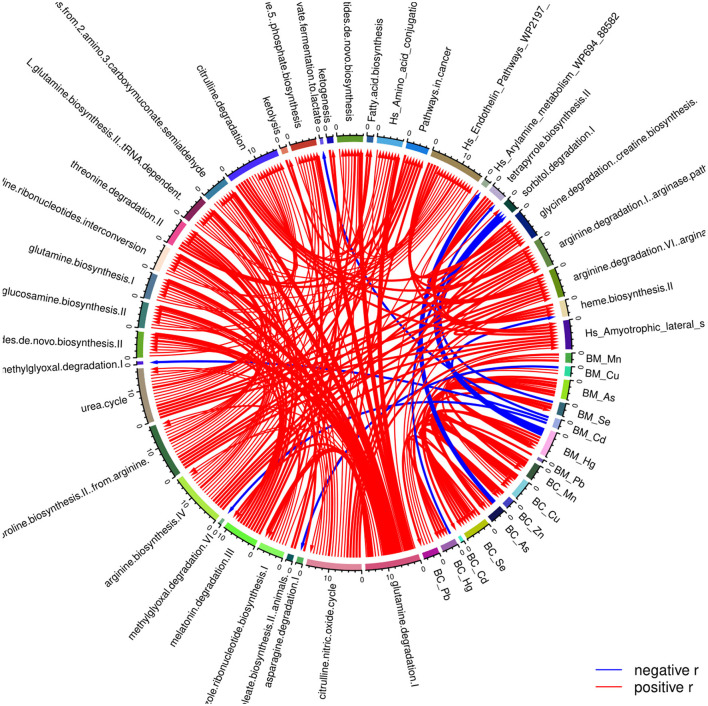
Correlation globe of the exposure parameters and the pathways resulted from the detected metabolites in the PHIME participants. Prenatal concentration of Hg is negatively associated with the metabolic pathway sorbitol degradation I, while postnatal exposure to As is significantly associated with the metabolism of arylamine.

In addition, the levels of the various exposure biomarkers have been associated with the respective metabolic pathways. We found that prenatal concertation of Hg is negatively associated with the metabolic pathway sorbitol degradation I. In contrast, postnatal exposure to As is significantly related to the metabolism of arylamine (Figure 4).

**Figure 5:**
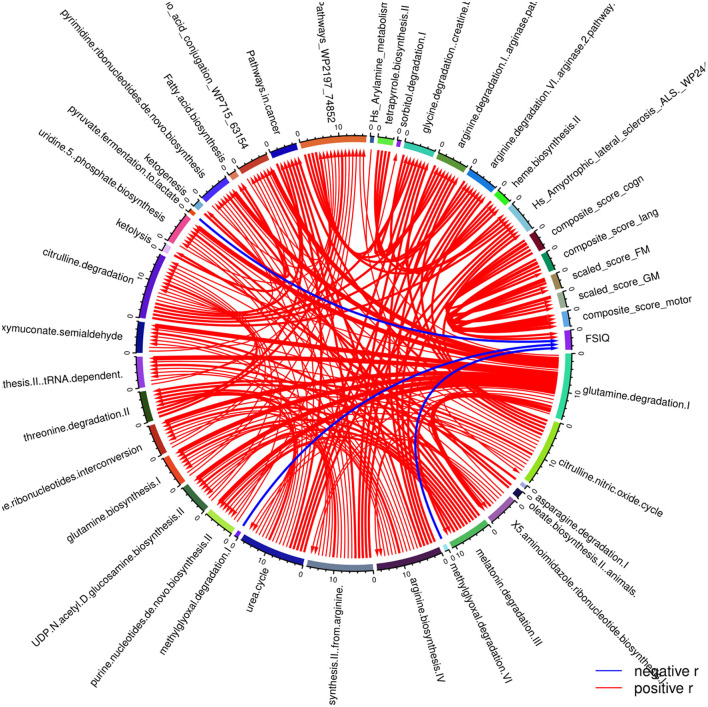
Correlation globe of the health outcomes regarding the neurodevelopment of the children participated in the PHIME cohort and the metabolic pathways. Cognitive capacity of the children (FSIQ) is negatively associated the following metabolic pathways: methylglyoxal degradation I, the methylglyoxal degradation VI and the pyruvate fermentation to lactate. In addition, motor development is significantly negatively associated with the urea cycle, the biosynthesis of arginine, the biosynthesis of proline from arginine, the degradation of citrulline and the citrulline nitric oxide cycle.

Beyond the exposure parameters, metabolic pathways have also been associated with neurodevelopmental outcomes (Figure 5). In this case, it was found that the ones that are negatively associated with the cognitive capacity of the children (FSIQ) are methylglyoxal degradation I, methylglyoxal degradation VI and pyruvate fermentation to lactate. Motor development is negatively associated with the expression profiles of the metabolites involved in the urea cycle, the biosynthesis of arginine and of proline from arginine, the degradation of citrulline and the citrulline-nitric oxide cycle.

**Figure 6:**
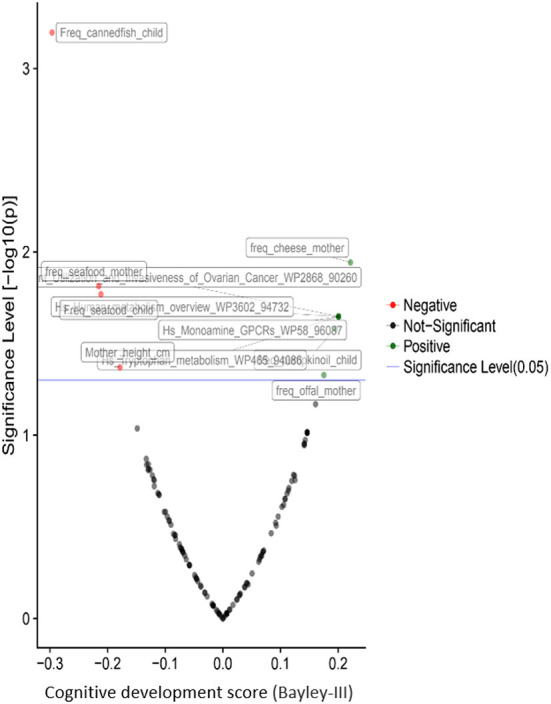
Volcano plot representing the correlations of children's cognitive development with prenatal and postnatal exposure factors during the first year of life based on the results from the PHIME cohort. The pathways resulted from the untargeted urinary metabolomics analysis. Cognitive development of children is positively associated with the metabolism of tryptophan indicating that the imbalance between the cellular reactive oxygen species (ROS), which may be an effect of exposure to metals, and the impossibility for the cell to detoxify them, leads to oxidative stress.

Child cognitive development is positively associated with the metabolism of tryptophan (Figure 6). According to the literature, abnormalities in the urea cycle and amino acid metabolism play a key role in adverse outcomes associated with oxidative stress (54, 55). The imbalance between the cellular reactive oxygen species (ROS), which may be an effect of exposure to metals, and the impossibility for the cell to detoxify them, leads to oxidative stress.

**Figure 7:**
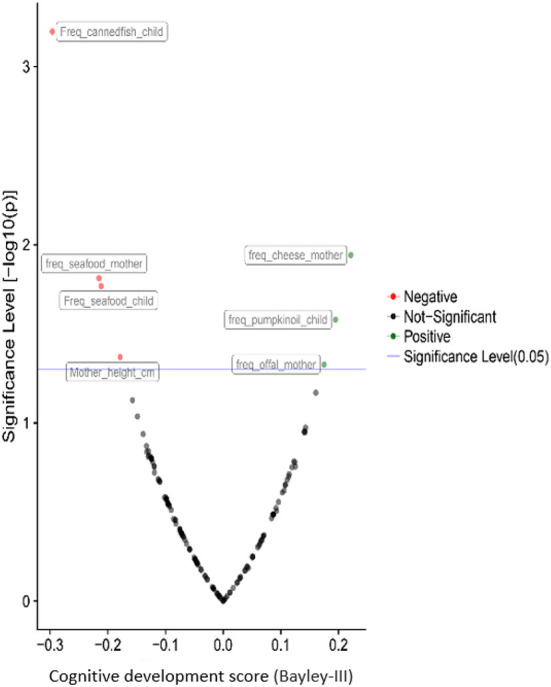
Correlations of children's cognitive development with prenatal and postnatal exposure factors during the second year of life based on the results from the PHIME cohort. The pathways resulted from the untargeted plasma metabolomics analysis. Besides the associations of psychomotor development of the child with the urea cycle and the amino acids metabolism, dietary and socioeconomic factors play a key role to child neurodevelopment.

Besides the associations of child psychomotor development with the urea cycle and the metabolism of the amino acid, dietary and socioeconomic factors play a crucial role in neurodevelopment. Cheese, offal and local food consumption during the pregnancy and pumpkin oil consumption from the child benefit the cognitive and gross motor development. In contrast, seafood consumption, especially canned fish, is negatively associated with cognitive (Figure 7) and fine motor development. The father's age is the only factor significantly associated with language development, but it was also negatively correlated with child motor development. As expected, the education of the mother's partner was positively correlated with the child's cognitive development.

### HERACLES cohort

**Figure 8:**
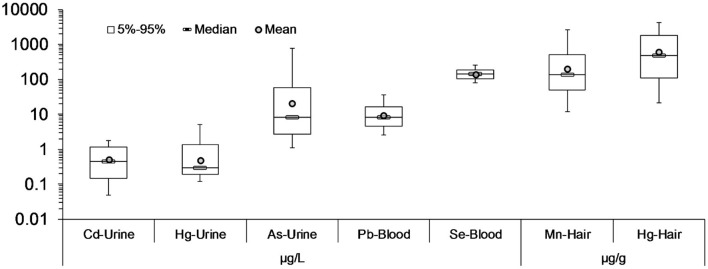
Heavy metals concentration in blood, urine and hair from the children participated in HERACLES cohort. In urine, As was found in highest concentrations, while in hair the Hg. The lowest concentrations in urine belongs to Cd, and in hair in Se, which role is mostly beneficial for a child's neurodevelopment.

The levels of heavy metals were monitored in both environmental (soil) and biological matrices. On top of that, dietary and sociodemographic data had also been collected. The corresponding biomonitored levels are illustrated in Figure 8. In urine, As was the most abundant, while Hg was found in hair. The lowest concentrations in urine corresponded to Cd and in hair to Se—it is noteworthy that Se is primarily beneficial to child neurodevelopment at these concentrations.

**Figure 9:**
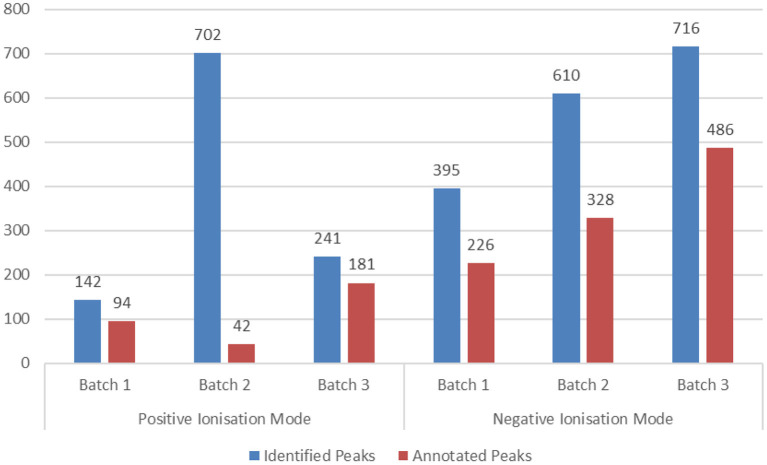
Metabolomics analysis results per analyzed batch and ionization mode of the HERACLES cohort. The samples were analyzed in three different analytical batches due to technical limitations of the used platform. At the end of each batch, the system was cleaned and re-calibrated to avoid the introduction of variations. In total, 2,806 peaks were identified, while 1,742 were annotated and used as input for the pathway analysis.

Metabolomics analysis resulted in the detection of 2,806 peaks. Sixty-two percentage of the detected peaks were annotated. Most detected metabolites correspond to carboxylic acids and derivatives and, more specifically, to amino acids, peptides, and analogs (Figure 9).

**Figure 10:**
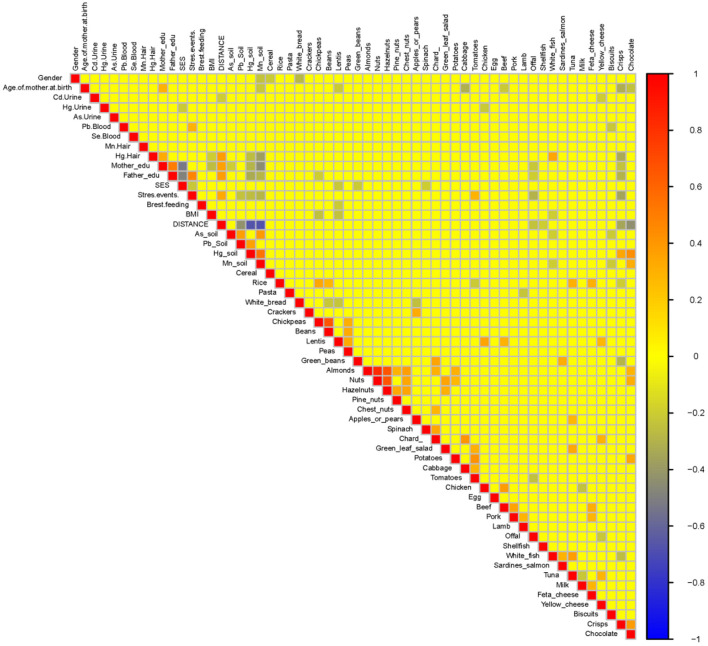
Exposure parameters heatmap for the HERACLES cohort. The distance of the living address found to be highly negatively associated with the concentrations of Hg and Mn in soil. In addition, white fish consumption is highly correlated with the concentrations of Hg in hair. The significance of these results is associated with the impact of exposure to Hg and Mn on children neurodevelopment. The exposure levels on Hg and Mn are correlated with most of the WISC IV indices.

**Figure 11:**
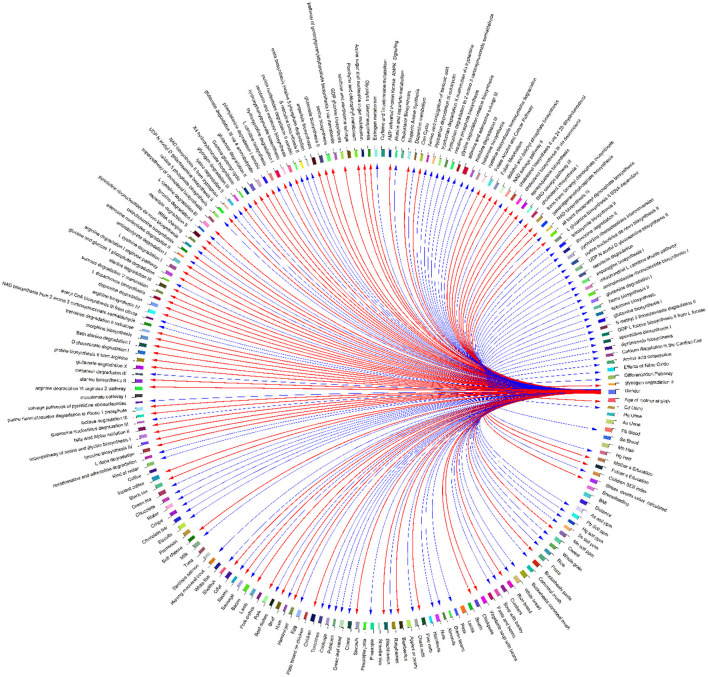
Correlation globe of exposure factors and detected metabolic pathways in HERACLES cohort participants. Based on the results metabolic pathways related to amino acids metabolism and urea cycle found to be highly associated with the concentrations of heavy metals in biological samples. For example, the biosynthesis of L-tyrosine found to be significantly correlated with the concentrations of Pb (*p*-value = 0.04), Mn (*p*-value = 0.02), and Hg (*p*-value = 0.03). The biosynthesis of NAD from tryptophan, and the synthesis of acetyl-CoA from citrate are highly correlated with the Pb concentrations (*p*-value = 0.02). Also, the metabolism of the neurotransmitter dopamine is correlated with the concentrations of Pb (*p*-value = 0.02), and Hg (*p*-value = 0.01). The correlations between the detected pathways related to amino acids metabolism and urea cycle and the concentrations of metals, indicate imbalance between the cellular reactive oxygen species (ROS), which may be an effect of exposure to metals, and the inability of the cell to detoxify them, leading to oxidative stress.

**Figure 12:**
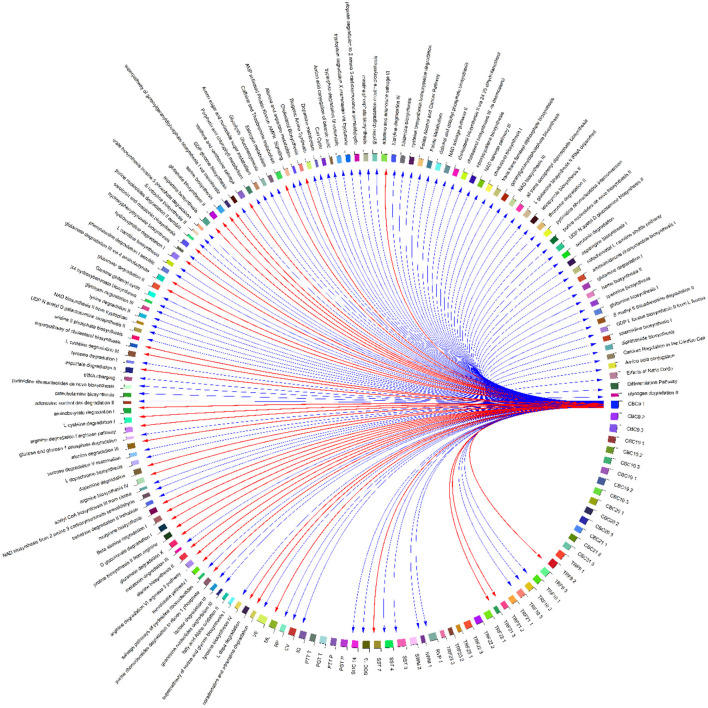
Correlation globe of health outcomes and detected metabolic pathways in HERACLES cohort participants. No significant relationships were found between exposure factors and TRF values, while the conjoint behavioral consultation (CBC) indices and metabolic pathways related to urea cycle were highly correlated (for example biosynthesis of aspartate had a positive relationship with the Attention Deficit/Hyperactivity Problems index (*p*-value = 0.02).

**Figure 13:**
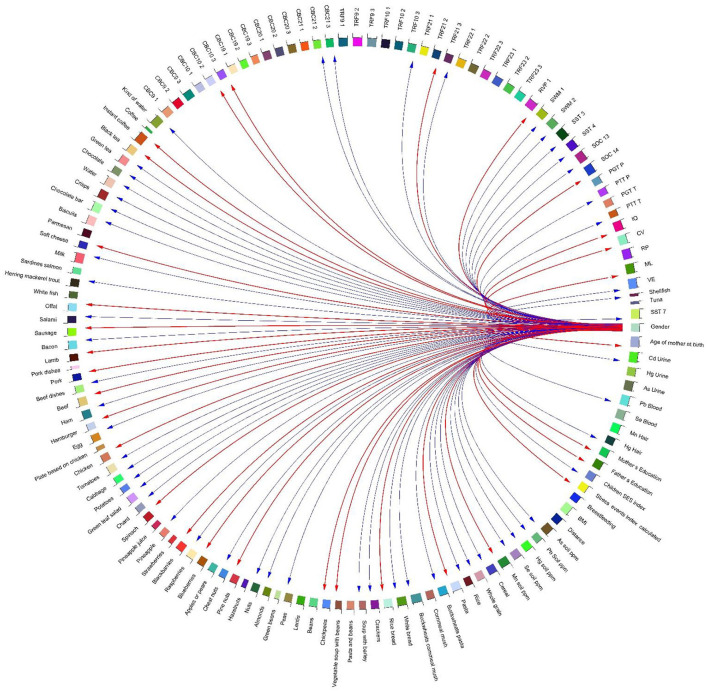
Correlation globe of exposure factors and health outcomes for the children participated in HERACLES cohort. Our study revealed significant associations between the conjoint behavioral consultation (CBC) indices and exposure to metals [for example the Attention Deficit/Hyperactivity Problems index was negatively correlated with exposure to Hg (*p*-value = −0.03)]. No significant relationships were found between exposure factors and TRF values.

A heatmap (Figure 10) and three correlation globes (Figures 11–13) have been produced to understand better how the various parameters are correlated.

The distance of the residential address from the contamination source has been identified as a determinant for most of the WISC IV indices. At the same time, distance from the source is highly negatively associated with the concentrations of Hg and Mn in soil (Figure 10). In addition, our study revealed significant associations between the conjoint behavioral consultation (CBC) indices and exposure to metals [for example, the Attention Deficit/Hyperactivity Problems index was positively correlated with exposure to Hg (*p*-value = 0.02)] (Figure 13).

**Figure 14:**
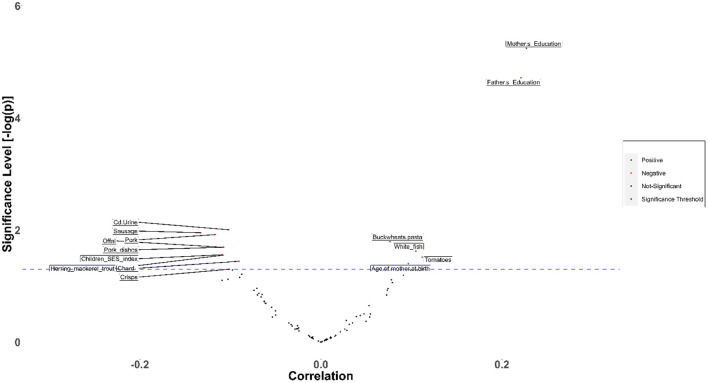
Association of intelligence quotient with environmental, dietary and exposure factors in the framework of HERACLES cohort. Based on the analysis, distance of the living address has been pointed out as a key determinant for most of the WISC IV indices. With regard to the dietary factors, it was found that inclusion of tomatoes often in the diet, has a beneficial impact on IQ, Verbal Comprehension index and Working Memory. At the same time, cereal rich diets, are highly associated with the Perceptual Reasoning index. It has also to be noted that both tomatoes and cereals are beneficial for cognition indices. White fish has been found to be associated with lower score in Social Responsiveness Scale thus, to provide protection against ASD impairments, in contrast to other food items such as pork, sausages, crisps, herring mackerel, and chards that impact adversely social responsiveness. Besides nutrition, social factors, play an important and crucial role.

Regarding dietary factors, it was found that the inclusion of tomatoes often in the diet has a beneficial impact on IQ (Figure 14), Verbal Comprehension index and Working Memory. At the same time, cereal-rich diets are highly associated with the Perceptual Reasoning index. Overall, regular consumption of tomatoes and cereals has a beneficial effect on cognition indices. Based on the conclusions of previous studies, the positive effect of tomatoes is mainly attributed to the antioxidant activity of lycopene, which has been proven beneficial for a broad variety of adverse outcomes, both mental (e.g., psychiatric disorders) and physical (CVD, cancer) (56). Cereal consumption during breakfast has been proven in many studies a significant booster to cognitive abilities relevant to performance in educational activities (57).

In addition, tomatoes, white fish, nuts and raspberries are associated with a lower score in Social Responsiveness Scale, indicating that regular consumption of this foodstuff may have a protective role against ASD impairments, in contrast to other food items such as pork, sausages, crisps, herring mackerel and chards that affect social responsiveness adversely.

Fish is generally considered among the most valuable food items concerning child neurodevelopment. However, it has been shown that this is related to (a) fish type and (b) the specific neurodevelopmental parameter under investigation. More precisely, it has been found that white fish consumption is positively associated with cognitive development, while it is not beneficial on indices related to Perceptual Reasoning and Working Memory. It is worth mentioning that according to our results, white fish is also associated with the concentrations of Hg in hair. The associations of white fish consumption, Hg concentrations, and neurodevelopment must be further investigated to help the authorities establish the most beneficial public health advice regarding consumption of white fish.

Besides nutrition, social factors play an essential role on child neurodevelopment. Factors negatively correlated with neurodevelopment were maternal partner's age, maternal weight, and the source of drinking water. On the other hand, maternal and paternal education were positively associated with IQ, as expected from already published studies (58).

Based on the results, metabolic pathways related to amino acid metabolism and the urea cycle are highly associated with heavy metals concentrations in biological samples. For example, the biosynthesis of L-tyrosine was found to be significantly correlated with the concentrations of Pb (*p*-value = 0.04), Mn (*p*-value = 0.02), and Hg (*p*-value = 0.03). The biosynthesis of NAD from tryptophan and the synthesis of acetyl-CoA from citrate are highly correlated with the Pb concentrations (*p*-value = 0.02). Also, the metabolism of the neurotransmitter dopamine is correlated with the concentrations of Pb (*p*-value = 0.02) and Hg (*p*-value = 0.01). The correlations between the detected pathways related to amino acids metabolism and urea cycle and the concentrations of metals indicate an imbalance between the cellular reactive oxygen species (ROS), which may be an effect of exposure to metals, and the inability of the cell to detoxify them, leading to oxidative stress (Figures 11, 12).

Moreover, cohort participants identified specific metabolic signatures in children diagnosed with Autism spectrum disorder (ASD). As and Hg exposure levels were above the 95th percentile. According to untargeted metabolomics analysis, the levels of L-arginine and L-tryptophan were above the 95th percentile, while (S)-lactate was below the 5th percentile. The results mentioned above suggest significant disturbances in cell biochemistry, which resulted in the impairment of antioxidant defense mechanisms leading to the clinically observed results in linguistic, motor development and cognitive capacity.

## Discussion

Exposome science provides opportunities for a paradigm shift that has the potential to provide a more in-depth understanding between environmental exposure and clinical outcomes. Studying the exposome allows us to capture the complex interactions resulting from both complex physical, chemical and biological exposures, and dietary and sociodemographic factors during the human lifespan (including *in utero* life). Linking this comprehensive view of exposure determinants and modulators and of the corresponding exposure levels with the underlying biology we can connect mechanistically and dynamically in time exposure triggers and molecular mechanisms related to adverse health outcomes, through multi-omics, bioinformatics and systems biology analysis.

The mechanisms involved in impaired child neurodevelopment based on our results, namely perturbations in the citric acid cycle, urea cycle, and amino acid metabolism, are proven to be significant in the oxidative stress cascade. Formate, 2-oxoglutarate, isocitrate, glycerol, carnitine, glutathione, methionine, cysteine, pyruvate, N-acetylglutamic acid, β-alanine, serine, arginine, citrulline, tryptophan, alpha-D-glucose, (S)-lactate and acetate, which have been detected in samples from the above cohorts, could be candidate biomarkers for neurodevelopmental disorders related to oxidative stress.

Based on data from high dimension biological analysis, exposure to heavy metals results in abnormal mitochondrial function. Considering the importance of mitochondria in energy metabolism and cellular signaling, along with the observed disruption in glycolysis, it is of no surprise that an association between exposure to heavy metals and impaired neurodevelopment is found. More specifically, in both studies related to child neurodevelopment, it was found that impaired energy production due to environmental factors at an early developmental stage, be that prenatal or postnatal, is crucial for child neurodevelopment. In addition, perturbations of the identified pathways, for the homeostatic operation of which the presence of the above biomarkers is crucial, must be examined as a putative underlying mechanism. Some of the identified pathways are S-methyl-5-thio-alpha-D-ribose 1-phosphate degradation, folate metabolism, serotonin degradation, taurine biosynthesis, citrulline-nitric oxide cycle, etc. Dysfunctions in carnitine metabolism may affect calcium homeostasis, which is involved in oxidative phosphorylation, leading to neurodevelopmental disorders. Biochemical markers directly or indirectly related to mitochondrial dysfunction, which were found to participate in dysregulated metabolic pathways were carnitine, alanine, lactate, pyruvate, lysine and acylcarnitine.

A critical methodological finding of the HERACLES study is that the simultaneous evaluation of environmental, sociodemographic and dietary parameters gives a more comprehensive picture of the most influential factors related to neurodevelopment impairment. Among these factors, distance of residential address to the primary contamination source in the area (landfill), an inverse proxy of environmental exposure, has been proven to be of key importance, followed by the effect of parental education level and child/family socioeconomic status.

Dietary components affect positively or negatively specific neurodevelopmental indices. Regular consumption of white fish and tomatoes and breastfeeding are positively associated with healthy neurological development during childhood. The high content of omega 3 fatty acids, which are considered as “brain foods” through neurotransmission regulation explains the positive effect of white fish. Omega 3 fatty acids benefit neurodevelopment mainly by modulating membrane biophysical properties and presynaptic vesicular release of classic amino acid and amine neurotransmitters (59). Tomatoes, too, have been recognized as solid antioxidants (60), being able to defend against the presence of reactive oxygen species (such as the ones generated by heavy metals) that can impair the mitochondrial function of neuronal cells (61).

Overall, the detailed dietary data collected in this study resulted in a more comprehensive interpretation of the interplay among exposure, dietary and sociodemographic parameters on child neurodevelopment. This, in turn, highlights why the exposome is a powerful tool for assessing the interaction between environment and health; it allows us to understand the actual exposure determinants (including environmental quality, lifestyle, diet and sociodemographics), which trigger biological responses related to adverse health effects; this enables to design targeted interventions toward protecting and promoting public health.

## Conclusions

Two are the main characteristics that stand out from these studies:

(a) We have chosen to consider factors such as dietary habits and socio-economic status as additional parameters associated with child neurodevelopment beyond environmental exposure to neurotoxicants; and

(b) We were particularly interested in capturing the overall metabolome perturbation and associating it with the totality of environmental exposure factors we could quantify on the one hand and with clinically observed neurodevelopmental disorders on the other. The aim of this coupled approach is to identify the metabolic mechanisms that govern the interactions between the exposome and the observed adverse health outcomes in order to support the built-up of adverse outcomes networks on the basis of real cohort data.

Thus, although the two studies included in this work involved different cohorts, the reanalysis of samples following a biology-based perspective allowed us to investigate how perinatal and early-life exposome affected child neurodevelopment. Finding associations between clinical or sub-clinical health outcomes and key features of the early-life external and internal exposome supports the elucidation of the mechanisms through which xenobiotics interact with and eventually perturb cell metabolism to induce specific pathways of toxicity in infants and young children. As a next step, *in vitro* testing coupled to targeted metabolomics on metabolically active relevant cell lines are planned to provide mechanistic evidence of the observed exposome-wide associations, building thus the evidence base for the development of the respective adverse outcome networks enhanced by exposome-wide analysis.

## Data availability statement

All the acquired data from both LCMS and NMR analysis were deposited to the EMBL-EBI MetaboLights database with the MTBLS1882 identifier.

## Ethics statement

The studies involving human participants were reviewed and approved by Aristotle University Ethics Committee. Written informed consent to participate in this study was provided by the participants' legal guardian/next of kin.

## Author contributions

OA has done the bioinformatics, the EWAS analysis for the HERACLES cohort, and was the one who had the responsibility for writing the manuscript. NP has carried out the bioinformatics and EWAS analysis for the PHIME cohort. CG, VD, and MD have carried out the metabolomics analysis. AK has worked on the EWAS analysis for the HERACLES cohort. IP has worked on the EWAS analysis for the PHIME cohort. MH has conceptualized and was responsible for the PHIME cohort in Slovenia, assisted by JS, who was also responsible for the heavy metal analysis, and the analysis of questionnaires. AT was responsible for the analysis of biomarkers in the HERACLES study. SK was responsible for the interpretation of the data. DS has conceived the exposome connectivity concept, was responsible for the study design, contributed significantly to the manuscript preparation, and internal review and revision. All authors contributed to the article and approved the submitted version.
